# *Bombyx mori* and *Aedes aegypti* form multi-functional immune complexes that integrate pattern recognition, melanization, coagulants, and hemocyte recruitment

**DOI:** 10.1371/journal.pone.0171447

**Published:** 2017-02-15

**Authors:** Dennis R. Phillips, Kevin D. Clark

**Affiliations:** 1 Department of Chemistry, University of Georgia, Athens, Georgia, United States of America; 2 Department of Food Science and Technology, University of Georgia, Athens, Georgia, United States of America; Uppsala Universitet, SWEDEN

## Abstract

The innate immune system of insects responds to wounding and pathogens by mobilizing multiple pathways that provide both systemic and localized protection. Key localized responses in hemolymph include melanization, coagulation, and hemocyte encapsulation, which synergistically seal wounds and envelop and destroy pathogens. To be effective, these pathways require a targeted deposition of their components to provide protection without compromising the host. Extensive research has identified a large number of the effectors that comprise these responses, but questions remain regarding their post-translational processing, function, and targeting. Here, we used mass spectrometry to demonstrate the integration of pathogen recognition proteins, coagulants, and melanization components into stable, high-mass, multi-functional Immune Complexes (ICs) in *Bombyx mori* and *Aedes aegypti*. Essential proteins common to both include phenoloxidases, apolipophorins, serine protease homologs, and a serine protease that promotes hemocyte recruitment through cytokine activation. Pattern recognition proteins included C-type Lectins in *B*. *mori*, while *A*. *aegypti* contained a protein homologous to *Plasmodium*-resistant LRIM1 from *Anopheles gambiae*. We also found that the *B*. *mori* IC is stabilized by extensive transglutaminase-catalyzed cross-linking of multiple components. The melanization inhibitor Egf1.0, from the parasitoid wasp *Microplitis demolitor*, blocked inclusion of specific components into the IC and also inhibited transglutaminase activity. Our results show how coagulants, melanization components, and hemocytes can be recruited to a wound surface or pathogen, provide insight into the mechanism by which a parasitoid evades this immune response, and suggest that insects as diverse as Lepidoptera and Diptera utilize similar defensive mechanisms.

## Introduction

Insects provide numerous benefits to society, but also exact an enormous toll in disease transmission and crop damage. Their evolutionary success has in part depended on the development of a sophisticated immune system that can repair wounds and destroy numerous pathogens. To be effective, an immune response requires a rapid and multi-faceted mobilization of hemolymph components, some of which are systemic, such as the production of antimicrobial peptides, while others, such as melanization, coagulation, and hemocyte encapsulation, must be localized to avoid harm to the host [1–4]. Melanization can be induced by damaged endothelial tissue [5], or by the recognition of microbial-derived pathogen-associated molecular patterns (PAMPs) by specific binding proteins present in the hemolymph [1]. This interaction triggers the sequential activation of a series of proteases that culminates in the cleavage of prophenoloxidases (PPOs) to phenoloxidases (POs) by PPO-activating proteases (PAPs), a process referred to as the PO cascade. POs can oxidize diphenolic compounds such as dopamine (DA) and L-dihydroxyphenylalanine (DOPA) to form melanin, but melanization only commences in Lepidoptera when the POs are incorporated into a high mass melanization complex that is capable of metabolizing the physiological substrate tyrosine (Tyr) [5, 6]. Whether this also occurs in other insect orders is not known. Melanization also requires the presence of accessory proteins called serine protease homologs (SPHs). In *Manduca sexta*, ProSPH1 and ProSPH2 are processed by proteases within the PO cascade, and then interact with PAPs and POs to greatly increase PO activity [7, 8]. The importance of melanization in immunity is underscored by the efforts that pathogens employ to inhibit the process. Of primary importance to this study is the endoparasitoid wasp *Microplitis demolitor*, which can propagate on numerous Lepidopteran hosts [9]. Oviposition by *M*. *demolitor* is accompanied by co-injection of a polydnavirus (PDV), which infects numerous host tissues, including hemocytes. PDV-infected cells express a number of viral gene products, including two Egf proteins (Egf1.0 and Egf1.5) that block melanization [10–12]. Both contain an N-terminal catalytic domain (CD) and a C-terminal repeat domain (RD) that function together to inhibit the PO cascade and prevent formation of the high mass melanization complex.

Coagulation is another important immune response that has been studied in numerous insect species [13]. Many coagulants have been identified, particularly in *Drosophila* [3], but little is known concerning how coagulation is initiated. Common coagulants include apolipophorins (ApoLp-I, -II and–III), hemolectin (homologous to hemocytin), and hexamerins (storage proteins (SPs)). Coagulant formation is stabilized by the activity of transglutaminases (TGs), which are Ca^2+^-dependent enzymes that cross-link proteins through the formation of an amide bond between specific Gln and Lys side chains [14]. Although TG activity associated with clotting has been demonstrated in *Drosophila* by using the artificial TG substrate biotin-cadaverine (B-cad), the specific TG that catalyzes this reaction has not been identified. In contrast, targets of this TG have been identified in *Drosophila* hemolymph by B-cad incorporation, including ApoLps, hexamerins, PO, and pathogen surfaces [14].

Hemocyte encapsulation as a localized immune response has been studied extensively in insects [15–19]. In Lepidoptera, capsules are comprised of two hemocyte subclasses, granular cells and plasmatocytes, which can recognize and attach to non-self surfaces [20]. Plasmatocytes subsequently spread and migrate in sufficient numbers to completely engulf and seal the target, a behavior induced by the cytokine plasmatocyte spreading peptide (PSP), a member of the large Lepidopteran-derived ENF-peptide family named for their invariable N-terminal ENF sequences [4, 21–23]. Although putative ENF homologs have been identified in mosquitos, they have not yet been functionally characterized [24]. Immune activation or wounding results in the cleavage of ENF peptides from the C-terminus of a larger inactive propeptide during an immune response [25], but neither the identity of this protease, its mode of activation, or its localized production are understood.

Here we propose that melanization, coagulation, and encapsulation are functionally integrated into a large protein array that we call the Immune Complex (IC). We have taken a top-down approach to study IC formation by using innovations in mass spectrometry (MS) instrumentation and proteomics methodology to identify IC components. We have also designed and synthesized a novel affinity-labeled cross-linking substrate to efficiently identify specific targets of TG. Our findings in both *B*. *mori* and *A*. *aegypti* indicate that the IC contains all the components necessary to fulfill the functions of a localized immune response, including recognition proteins for targeting the IC to non-self surfaces, the enzymatic machinery to form melanin, an array of coagulant proteins, and a cytokine-producing serine protease (SP) involved in hemocyte recruitment. We also show that the potent melanization inhibitor Egf1.0 not only blocks the incorporation of multiple proteins into the *B*. *mori* IC, but also inhibits TG-induced cross-linking. These novel findings demonstrate the immune-induced assembly of higher-order multi-functional structures across different insect orders, and provide evidence of the cross-talk that occurs between immunity and coagulation. We also show that, like Lepidoptera, *A*. *aegypti* POs are not metabolically active toward the physiological substrate Tyr, and that additional processing and/or IC incorporation is required, adding further proof that the long-standing PO cascade paradigm of proteolytic PO activation is untenable in its current form.

## Results

### Immune complex proteins in *B*. *mori* and *A*. *aegypti*

**Fig 1:**
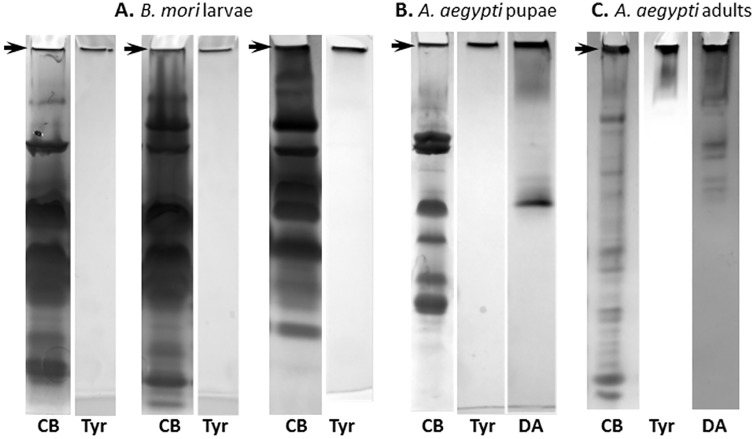
Tyr-dependent melanization in *B*. *mori* and *A*. *aegypti*. **A.** *B*. *mori* plasma was prepared from three larvae and separated in duplicate by native-PAGE. Proteins were stained with Coomassie Blue (CB) and melanizing activity was measured by the addition of Tyr. The arrows indicate stained bands that were excised for analysis by LC-MS/MS. **B.** Plasma from twenty *A*. *aegypti* pupae was separated by native-PAGE and stained with Coomassie Blue (CB), Tyr, or DA. **C.** Plasma from twenty *A*. *aegypti* adult females also separated by native-PAGE and stained as in B. The arrows indicate stained bands that were excised for analysis by LC-MS/MS.

To isolate and identify components of the IC, plasma samples were prepared from 5^th^ instar *B*. *mori* larvae, separated by native-PAGE, and stained for protein and Tyr-dependent melanization (Fig 1A). Although the larvae exhibited variable protein patterns, Tyr-dependent melanization occurred solely in a narrow band at the gel/well interface. The corresponding Coomassie Blue-stained bands were excised, digested, and analyzed by LC-MS/MS. The large array of proteins identified were classified by likely primary function, including melanin production, coagulation, pattern recognition, and enzymatic activity (Table 1). PO-cascade-derived proteins included the enzymes PO1 and PO2, and the non-enzymatic SPH1 and SPH2. Coagulation-related proteins included Apo-Lps I, II, and III, as well as the large multi-domain hemocytin protein. Three different storage proteins were also identified. We tentatively classified these as coagulants given their propensity to form hexamers and larger order multi-hexamers, and their numerous reported immune functions [26–30]. Pattern recognition proteins with likely immune functions were also found, including two lectins, C-type 19 and C-type 21, and multiple members of the large 30 kD lipoprotein family (Bm Lp2, Lp4, Lp5, Lp6, and Lp15), none of which have been previously recognized as forming high mass aggregates. We also found two proteins with potential enzyme activity, a carboxylesterase (CE) and a serine protease (SP3), and a protease inhibitor, serpin 9.

**Table 1:** *B*. *mori* IC components identified by LC-MS/MS.

Accession #	Description	ΣCoverage	Σ# Proteins	Σ# Unique Peptides	Σ# Peptides
**Coagulants**					
347543546	Apolipophorin I/II	61.83	1	171	171
112983018	Apolipophorin III	40.32	2	9	9
162462371	Hemocytin	15.07	1	37	38
1335609	Storage-protein 1	39.75	3	28	28
124430725	Sex-specific storage-protein 2	22.19	1	11	11
379327811	Silkworm storage protein	11.78	2	5	5
**PO Cascade**					
112983667	Phenoloxidase 1	35.04	2	18	18
163838668	Phenoloxidase-2	44.59	1	5	24
112983448	“	48.63	1	4	23
112983100	Serine protease homolog 1	19.05	1	7	7
114052256	Serine protease homolog 2	7.02	1	4	4
**Pathogen Recognition**					
284813581	C-type lectin 19	11.78	1	3	3
112983062	C-type lectin 21	17.57	1	5	5
1335608	Bm Lp2, Lp4, Lp5, Lp6, Lp15	58.47	2	1	11
379046488	“	47.35	1	3	10
126419	“	44.70	2	3	10
266439	“	39.92	1	7	9
**Enzymes**					
114050871	Carboxylesterase clade H	13.31	1	5	5
114050919	Clip domain serine protease 3	8.74	2	3	3
**Serpin**					
14028769	Serpin 9	8.10	2	2	2

Samples were processed as described in the Material and Methods. Search parameters were set at a high confidence with a Target of 0.05, which produced an Actual Relaxed False Discovery Rate of 0.0496. A minimum of two peptides were required for a protein match. This list was ordered to classify identified proteins according to their activation source (PO cascade) or their likely primary immune function. Obvious exogenous (trypsin) and endogenous contaminants (imaginal disk growth factor, heat shock protein and sericin) were removed. Duplications were also noted (PO2) and numerous proteins were renamed for ease of identification. Identification of specific 30 kD Lp proteins was problematic due to ambiguity in the NCBI and SilkDB databases. The Mascot search recognized four different 30 kD Lp proteins in the NCBI database. These were blasted against the silkworm protein database (www.silkdb.org) to cross-reference the nomenclature. Due to the high degree of homology, each recognized a set of five related sequences. These sequences were renamed according to Zhang et al. [31] and are listed as a group. All five belong to cluster III within the typical 30 kD Lp family [31, 32]. The chymotrypsin inhibitor was renamed according to Zou et al. [33].

Previous studies have shown that mosquitos express and utilize a set of immune proteins similar to those found in *B*. *mori* and *M*. *sexta* [34], but whether Tyr-dependent melanization and IC formation occur is unknown. Plasma prepared from *A*. *aegypti* pupae and separated by native-PAGE exhibited a large number of proteins, yet when incubated with Tyr showed only a heavily melanized band at the gel/well interface, indicating the presence of POs (Fig 1B). We repeated this experiment using plasma from adult females, which exhibited a protein staining pattern distinct from pupae, but still melanized strongly at the gel/well interface in the presence of Tyr (Fig 1C). In contrast to Lepidoptera, *A*. *aegypti* contains ten PPO genes, each of which can exhibit differential expression and/or immune activation. To determine whether other POs were present, we incubated the native-PAGE-separated pupal and adult proteins with dopamine, which POs can metabolize to form melanin. Dopamine staining indicated that a large number of additional activated POs were present that were unable to metabolize Tyr and were not incorporated into the IC (Fig 1B and 1C). To identify IC proteins in *A*. *aegypti*, we excised and analyzed by tandem MS the bands at the gel/well interface (Table 2). In common with *B*. *mori*, the IC contained two POs (PPO1, PPO3), Alp, and SPHs (S6 Fig). There were also important differences not only with *B*. *mori*, but also between *A*. *aegypti* pupae and adults. Although both pupal and adult ICs contained SPHs, pupae contained three, while adults contained two, with only one common SPH between them. Also, the pupal IC, like *B*. *mori*, contained multiple hexamerins, but none were observed in the adult IC. Pupae and adults also exhibited very different patterns of Leucine-rich-repeat (LRR) proteins. Like *B*. *mori* SP3, adult females contained a SP (CLIPB34), which has no known function.

**Table 2:** *A*. *aegypti* IC components identified by LC-MS/MS. Samples were prepared and identified as in Table 1.

Accession # (VectorBase)	Description:	Pupae ΣCoverage	Adult ΣCoverage
**PPOs**			
AAEL013498	PPO1	11.99	27.63
AAEL011763	PPO3	5.82	24.71
**LRR Proteins**			
AAEL012086	LRIM1		26.91
AAEL009520	LRIM2		20.18
AAEL010128	LRIM4		18.65
AAEL001402	LRIM10B		4.95
AAEL000668	uncharacterized LRR	6.67	
**Hexamerins**			
AAEL013981	Hexamerin 2 beta	11.85	
AAEL000765	Hexamerin 2 beta	6.64	
AAEL008045	Hexamerin 2 beta	2.72	
**Serine Proteases/Homologs**			
AAEL002600	SPH	9.21	3.32
AAEL002629	SPH	8.56	
AAEL002595	SPH	6.31	
AAEL002610	SPH		3.82
AAEL000028	SP (CLIPB34)		5.80
**Apolipophorins**			
AAEL009955	Alp	52.24	35.47
**Other Proteins**			
AAEL008598	Vitellogenin-like	2.28	
AAEL006102	Gelsolin		31.36
AAEL009629	EndoU protein, XendoU domain		15.09
AAEL004156	Fibrinogen		14.34
AAEL001794	Macroglobulin		12.44
AAEL006962	Carbohydrate-binding domain		20.88
AAEL006434	polyserase 2-like		3.91

Endogenous contaminants such as ribosomal proteins derived from lysed hemocytes or other tissues were removed.

### Coagulation and melanization are physiologically associated

**Fig 2:**
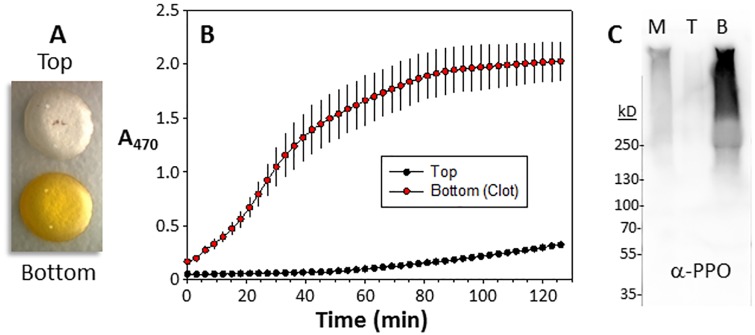
Tyr-dependent melanization is associated with coagulation in *B*. *mori*. **A.** Plasma was prepared and then centrifuged in a 10 kD cutoff filter, which separated the plasma into a clear top layer and a viscous coagulant-containing bottom layer. **B.** The layers were tested separately for Tyr-dependent melanizing activity. **C**. The layers were immunoblotted (T = Top, B = Bottom, and M = Mixed) using a *B*. *mori*-specific antibody to PPO.

The association of coagulants with melanization-related proteins in the native gels could represent a true physiological association, or a non-physiological trapping of two or more high mass complexes unable to enter the gel. We therefore used *B*. *mori* plasma, which coagulates and becomes viscous over time, to assess the association of coagulants and melanization components. We first incubated plasma until a coagulant formed, and then separated it from the remaining soluble proteins by centrifugation. Aliquots were removed from the top (non-viscous clear plasma) and the bottom (viscous pale yellow coagulant) of the tube (Fig 2A), and tested for melanization activity (Fig 2B) and the presence of PO (Fig 2C). Tyr-dependent melanization was found only in the coagulant-containing aliquot, and the majority of the PO, which was observed as a large high mass smear in a denaturing gel, was also associated with the coagulant. These results indicated that coagulant proteins, melanization-related proteins, and pattern recognition proteins are physiologically associated and interact to form a mature IC.

### Hemolymph TG stabilizes the IC

**Fig 3:**
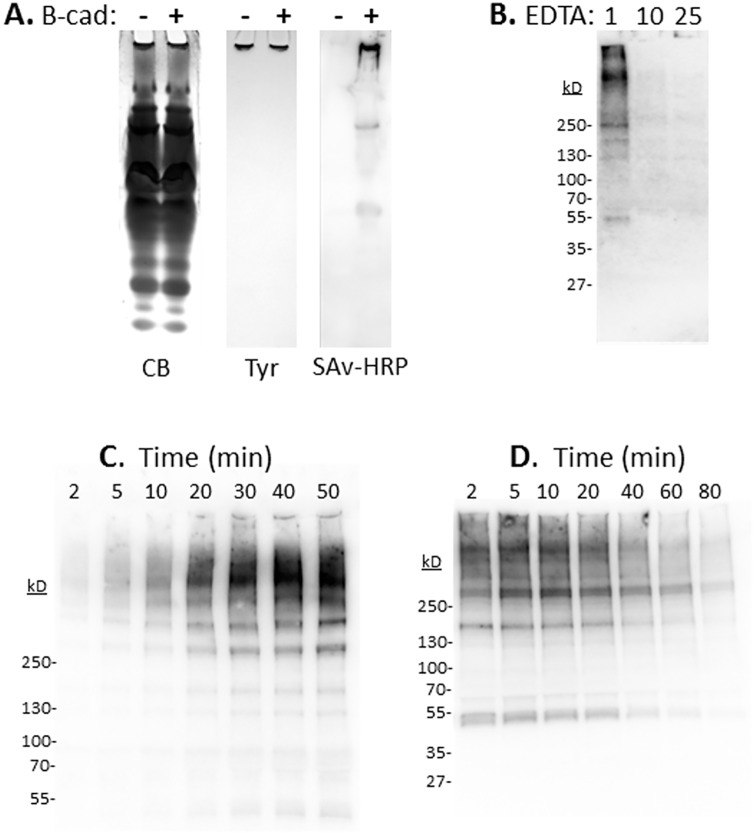
Characterization of the *B*. *mori* hemolymph TG activity. **A.** Plasma prepared from a single larva was separated into two aliquots, one of which was treated with 4 mM B-cad for 2 h at 22°C. The control and treated samples were analyzed in triplicate by native-PAGE, with one set stained for total protein (CB), another for Tyr-dependent melanizing activity, and the last affinity-blotted for the presence of biotin (SAv-HRP). **B.** Effect of EDTA on B-cad labeling. Plasma was prepared with 4 mM B-cad and the indicated concentrations (mM) of EDTA (Na_2_EDTA, pH 7.0), and then incubated at 22°C for 2 h. **C.** Time course of B-cad labeling. Plasma was prepared with 4 mM B-cad and aliquots were removed at the indicated times to determine the rate of protein labeling. **D.** Duration of TG activity. Plasma was prepared and B-cad was added at the indicated times. Samples from each time point were processed after a 1 h incubation at 22°C. All samples were separated by SDS-PAGE and affinity-blotted with SAv-HRP.

The inherent stability of the ICs suggests the presence of strong non-covalent interactions and/or covalent cross-linking between the IC components. To determine whether TGs are responsible for cross-linking components of the IC, we used the artificial TG substrate B-cad, which can substitute for Lys to produce biotin-labeled Gln residues [14]. *B*. *mori* plasma samples were incubated with and without B-cad for 1 hour and then analyzed by native-PAGE for total protein, Tyr-dependent melanization, and biotin labeling (Fig 3A). Protein staining and melanizing activity were unaffected by B-cad addition, indicating that B-cad incorporation was not extensive enough to destabilize the IC. In contrast, only the sample containing B-cad exhibited a strongly affinity-labeled band at the gel/well interface that was co-incident with the Tyr-staining. Two smaller bands were also noted on the gel in the B-cad sample, but their identity was not determined.

### Characterization of the hemolymph TG and identification of TG substrates

To determine whether B-cad labeling is catalyzed by a Ca^2+^-dependent TG, we prepared plasma samples with and without the chelator EDTA, and then separated them by SDS-PAGE. The control plasma contained numerous B-cad labeled proteins, while B-cad labeling was greatly diminished in the samples containing EDTA (Fig 3B). EDTA showed little inhibition of melanin production, suggesting that PO activity in the IC was still intact. To determine the rate of cross-linking by TG, we added B-cad immediately after plasma preparation, and then removed aliquots at the indicated times (Fig 3C). B-cad containing proteins were discernable by 2 min and maximum labeling was complete by 30 min. We then determined the duration of TG activity by preparing plasma and adding B-cad at the indicated times after bleeding (Fig 3D). Protein labeling with B-cad was observed up to 20 min post-bleeding, but declined in subsequent time points, suggesting inactivation of TG and/or substrate depletion.

Although incorporation of B-cad into the IC was sufficient to observe TG activity, attempts to affinity-purify B-cad-containing proteins led to low yields of identifiable substrates. We therefore designed and synthesized a series of novel TG substrates based on the tri-peptide Lys-Gly-Cys-Biotin (KGC-B), which more effectively mimics a natural target site within a protein (S1 Fig). Experiments designed to assess labeling efficacy showed KGC-B incorporation was greatly increased over B-cad (S2 Fig), while the N-terminal addition of a fluorescent tag provided another means of visualizing labeled proteins with no difference in labeling efficiency (S3 Fig). To identify specific TG substrates, we labeled plasma proteins with KGC-B and then affinity-purified proteins containing the Biotin tag. Separation by SDS-PAGE revealed a large number of proteins, with similar but not identical patterns when prepared with and without the reducing agent DTT (S4 Fig). LC-MS/MS analysis of the samples revealed a wide array of proteins targeted by plasma TG, most of which are immune-related (S1, S2 and S3 Tables). Surprisingly, a number of these proteins were found at multiple and unexpected molecular weights. Some were lower, which suggested proteolytic processing, while some were much higher, suggesting cross-linking with other proteins had already occurred. Some cross-linking may be due to disulfide formation in the DTT-free sample. Although it has been suggested that PO activity may be responsible for this cross-linking [35], this was prevented by the inclusion of PO inhibitors (GSH and PTU) in the plasma preparation [6]. Overall, these data suggest that TG is cross-linking the same set of proteins found in the IC.

### Egf1.0 inhibits the incorporation of specific IC components

**Fig 4:**
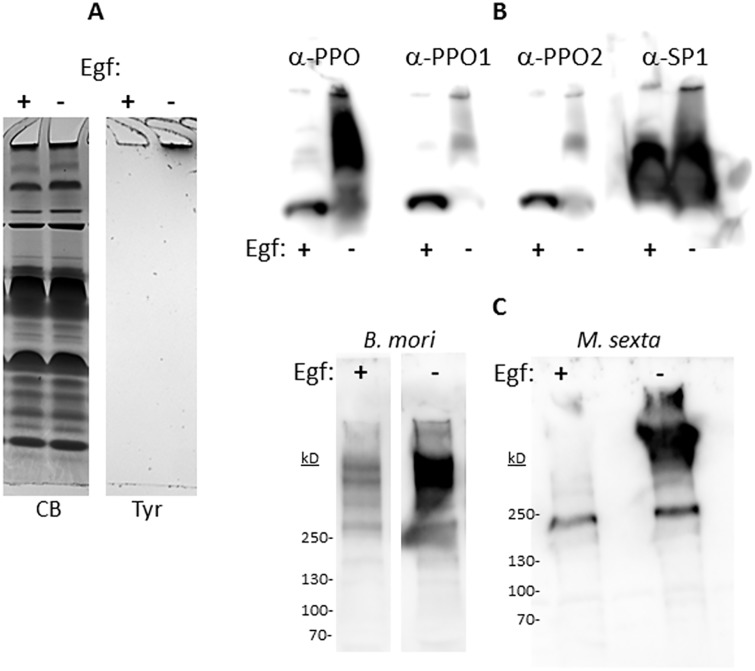
Egf1.0 inhibits melanization, IC formation, and TG activity. **A.** Plasma prepared from a single *B*. *mori* larva was separated into two aliquots, one of which was treated with Egf1.0 (200 μg/mL final concentration). After a 1 h incubation at 22°C, duplicate samples were run on native-PAGE, with one set stained for protein using Coomassie-Blue (CB), and the other with Tyr for melanization. **B.** Egf1.0 inhibits the inclusion of POs and SP1 into the IC. Plasma was prepared as in part A and separated by native-PAGE. Samples were immunoblotted with antibodies specific to *B*. *mori* PPO, PPO1, PPO2, and SP1. **C.** Egf1.0 inhibits B-cad protein labeling in *B*. *mori* and *M*. *sexta*. Plasmas were prepared with and without Egf1.0 (200 μg/mL) and B-cad (4 mM final concentration), incubated at 22°C for 40 min (*B*. *mori*) or 70 min (*M*. *sexta*), and then separated by native-PAGE. Samples were affinity-blotted with SAv-HRP.

The parasitoid wasp *M*. *demolitor* inhibits melanization in Lepidoptera through the action of virally-derived Egf proteins. While Egf1.0 and Egf1.5 are thought to block the proteolytic activation of PPOs by inhibition of PAPs, they likely possess additional functions [10–12]. For example, Egf1.0 has been shown to block melanization at the gel/well interface [6], and prevents its formation and attachment to solid surfaces (S5 Fig). To determine whether Egf1.0 affects the assembly of the other components within the IC, we collected plasma from a *B*. *mori* larva and immediately added Egf1.0 to an aliquot. Protein staining after native-PAGE separation was identical with or without Egf1.0, but only the untreated sample in the Tyr-stained gel melanized at the gel/well interface (Fig 4A). To show that Egf1.0 treatment blocked formation of the required enzymatic machinery, the bands at the Coomassie-stained gel/well interface were excised, digested, and subjected to LC-MS/MS to determine IC components (Table 3). Some proteins were unaffected by the addition of Egf1.0, including the Lectins, ApoLps, hemocytin, the storage protein SSP2, the serine protease homolog SPH2, the CE, and numerous 30 kD Lp proteins. Two additional proteins not detected in the experiment presented in Fig 1, the antimicrobial peptide attacin and the nuclopolyhedrovirus-associated promoting protein [36, 37], were also identified but were unaffected by the presence of Egf1.0. As expected, PO1 and PO2 were not detected in the Egf1.0-treated sample, and several other proteins were present at lower levels or absent, including SP1, a hexamerin normally associated with energy storage that is purported to have immune functions. Immunoblots of native-PAGE-separated proteins using PPO and SP1 antibodies confirmed the almost complete absence of IC-associated PPOs as well as a decreased amount of SP1 in the presence of Egf1.0 (Fig 4B). Other proteins absent in the Egf1.0-treated sample were two 30 kD Lp proteins, one of which, Lp3, was not detected in earlier experiments (Fig 1). Unlike SPH2, SPH1 was absent in the presence of Egf1.0, a surprising result given that both are thought to be activated through the PO cascade [38]. Finally, the protease SP3 was not detected. To date, no function has been assigned to SP3, yet its presence in the IC and its depletion by Egf1.0 suggests an immune-related function.

**Table 3:** *B*. *mori* IC components ±Egf1.0 identified by LC-MS/MS.

Accession #	Description	-Egf1.0 ΣCoverage	+Egf1.0 ΣCoverage
**Coagulants**			
347543546	Apolipophorin I/II	64.46	74.77
112983018	Apolipophorin III	43.01	60.75
162462371	Hemocytin	26.52	37.63
1335609	Storage-protein 1	14.21	--------
124430725	Sex-specific storage-protein 2	27.27	27.27
**PO Cascade**			
112983667	Phenoloxidase 1	31.68	--------
163838668	Phenoloxidase-2	45.74	--------
112983100	Serine protease homolog 1	23.10	--------
114052256	Serine protease homolog 2	37.59	43.61
**Pathogen Recognition**			
284813581	C-type lectin 19	22.61	25.48
112983062	C-type lectin 21	29.07	48.24
126419	Bm Lp2, Lp4, Lp5, Lp6, Lp15	39.39	25.00
266439	“	27.76	--------
293597264	Bm Lp3*	5.06	--------
**Enzymes**			
114050871	Carboxylesterase clade H	6.37	8.54
114050919	Clip domain serine protease 3	2.57	--------
**α-microbial**			
112983296	Attacin*	17.29	17.29
**Other**			
112984526	Promoting protein*	48.70	58.44
66527860	Egf1.0/1.5 (*M*. *demolitor*)*	--------	37.86

Samples were prepared and identified as in Table 1.

*Additional proteins not found in the data from Fig 1.

### Egf1.0 inhibits TG activity

Our data suggest that IC stability is provided through a combination of protein-protein interactions and TG-mediated cross-linking. Although Egf1.0 inhibited the inclusion of specific components into the IC, it left numerous coagulation and pathogen recognition components intact (Table 3). Because many of these proteins are substrates for TG (S4 Fig, S1–S3 Tables), we tested whether TG would continue to cross-link these components in the presence of Egf1.0. *B*. *mori* plasma was collected and added separately to solutions containing B-cad with and without Egf1.0. After incubation, samples were separated by SDS-PAGE and affinity-blotted with SAv-HRP to determine the presence of cross-linked Biotin (Fig 4C). Surprisingly, *B*. *mori* plasma in the presence of Egf1.0 exhibited a greatly reduced amount of biotin labeling, which was primarily present as a high mass smear in the control sample. We also tested TG-mediated cross-linking in *Manduca sexta*, which processes PPO and melanizes at a much slower rate than *B*. *mori* [5], and therefore is more effectively inhibited by Egf1.0. *M*. *sexta* plasma proteins from a 5^th^ instar day 1 larva [5] were also strongly labeled by B-cad, but Egf1.0 provided an almost complete inhibition of this labeling.

### The *B*. *mori* IC cleaves Pro-PSP to produce the immune cytokine PSP

**Fig 5:**
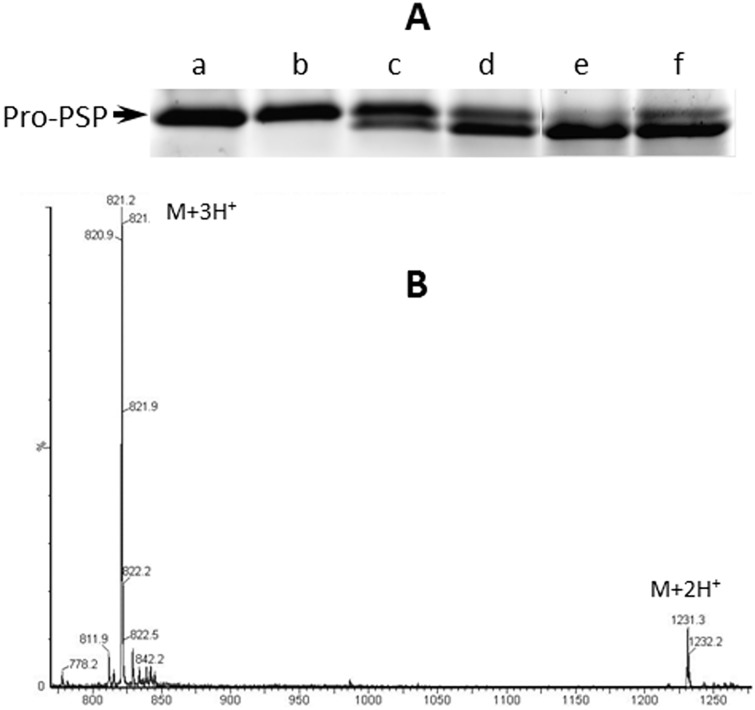
The IC correctly processes Pro-PSP. **A.** *B*. *mori* plasma was prepared and either separated on a native gel or washed free of low molecular weight substrates to make substrate-free plasma (SFP). IC-containing gel slices were excised as in Fig 1 and placed into tubes containing 25 μL of Pro-PSP (100 μg/mL) in TBS and incubated for 2 h at 22°C. Concurrently, equivalent Pro-PSP solutions were incubated with either 3 μL of plasma or 3 μL of SFP. Samples were separated by reducing SDS-PAGE and stained for protein with Oriole (Bio-Rad) to visualize Pro-PSP processing in: **a.** TBS; **b.** a control (non-IC containing) gel slice; **c.** an IC-containing gel slice (10 μL of plasma); **d.** an IC-containing gel slice (20 μL of plasma); **e.** plasma; **f.** SFP > 30 kD. **B.** ESI-QTOF MS of processed Pro-PSP. Pro-PSP solutions incubated with the IC-containing gel slices were tested for the presence of correctly processed PSP. The measured monoisotopic peaks (820.9 m/z [(M+3H^+^)^3+^] and 1230.7 m/z [(M+2H^+^)^2+^] matched the predicted mass (2459.1 Da (M)) of the disulfide-containing *B*. *mori* PSP (ENFVGGCATGFKRTADGRCKTYL).

Encapsulation and nodulation are immune responses that require both humoral and cellular components [39]. Binding of the IC to the surface of targeted pathogens is essential for a successful immune response, and may also be a prerequisite in recruiting hemocytes such as plasmatocytes and granular cells by the cytokine PSP. Despite intense interest, no one has yet identified its activating protease. Our own attempts to identify the protease indicated that the enzyme was part of a high mass protein complex, thereby preventing its isolation and identification. Identification of the putative SP3 protease in the IC led us to hypothesize that it may be the Pro-PSP activating protease. To test this, we excised the gel/well interface from native-PAGE-separated *B*. *mori* plasma and incubated it with *B*. *mori* Pro-PSP. After a 2 h incubation, the supernatant was removed and the proteins separated by SDS-PAGE for analysis. Both the Pro-PSP and a lower mass band were detected by Coomassie staining of the gel, suggesting that PSP had been cleaved from the pro-protein (Fig 5A). MS analysis confirmed the presence of a single peptide that was identical to the mass of correctly processed PSP (Fig 5B).

## Discussion

Here we present a unique analysis of high mass ICs from model species representing two insect orders, Diptera and Lepidoptera. Our initial studies in *B*. *mori* were the first to show that Tyr, long considered a poor substrate for POs, is the physiological substrate for melanization, and that its metabolism requires the incorporation of POs into a high mass protein complex. Here, we extend these results to show that the same metabolic requirement for melanization occurs in *A*. *aegypti*. In both species, POs are incorporated into ICs that form rapidly upon wounding and functionally integrate seemingly diverse responses that include pattern recognition, melanization, coagulation and hemocyte recruitment. In *B*. *mori*, we also show that the IC is extensively stabilized by TG-induced cross-linking, and provide insight into the mechanism by which IC formation is inhibited by pathogen-derived Egf1.0. A complete discussion of IC components in these two insects is not feasible; therefore, we will focus our discussion on the following key findings and the questions they raise:

*How does PO incorporation in the IC promote Tyr metabolism and melanization*? The PO cascade has provided a valuable framework for understanding the complexity of protein interactions required to initiate melanization, yet our demonstration of Tyr metabolism coupled to the incorporation of POs into the high mass IC indicates PAP cleavage of PPOs by itself is insufficient to provide full physiological activation. A number of PO activation mechanisms have been proposed, a detailed discussion of which can be found here [40]. Determining the actual mechanism requires further investigation, but we observed an interesting symmetry in the inclusion of two PO isoforms into the IC in both *B*. *mori* (*Bm*PO1 and *Bm*PO2) and *A*. *aegypti* (*Aa*PO1 and *Aa*PO3). This is even more remarkable in that *A*. *aegypti* expresses up to ten POs, many of which are expressed and metabolically active toward DA in both pupae and adults (Fig 1B and 1C) [41]. These results lead to important questions regarding the structural, functional, and physiological differences between the IC- and non-IC-associated *Aa*POs, and the similarities between *Aa*PO1/PO3 and *Bm*PO1/PO2 that lead to IC incorporation. We speculate that incorporation requires additional PO processing, which then leads to the formation of a defined higher order PO structure with the functional ability to metabolize Tyr. An example of additional PO processing that may be important for activity has been observed in Holotrichia [42]. Others have shown that members of the large Hexamerin family (POs and storage proteins) form multi-hexameric structures, but neither the cause nor the reason are known [28]. PO-dependent hexamer formation also likely requires assistance from “helper” proteins such as SPHs, which are known to promote DA-dependent PO activity and form high mass multimers in some insects [8, 43]. Interestingly, no other enzymes related to melanin production were found in the ICs. An obvious candidate is dopa decarboxylase, an enzyme commonly assumed to convert DOPA to dopamine during the process of melanization [44, 45]. Tyrosine hydroxylase has also been suggested as the first enzymatic step in the oxidation of Tyr [46], but its absence also shows that PO alone is sufficient. The role of dopachrome conversion enzyme in converting dopachrome to 5, 6-dihydroxyindole has also been suggested to be an important component in insect melanization [47], but we also found no evidence of this in the IC.

*What role do PRRs play in the IC*? The targeting of effector proteins to non-self surfaces requires pattern recognition receptors (PRRs), numerous examples of which are found in both ICs. We discovered similarities between the *B*. *mori* and *A*. *aegypti* ICs (ApoLps), as well as important differences (30 kD Lps, C-type lectins, hemocytin, LRIMs). The primary physiological role of ApoLps is thought to be lipid binding and transport, but numerous reports suggest immune functions, primarily through binding to membrane components from pathogens [48, 49], and their presence in high mass complexes [50–53]. In *Anopheles*, ApoLps have been shown to interact with the complement-like protein TEP1 [54], and to be involved in coagulation [50, 55]. However, most immune roles are associated with the myriad activities of ApoLp-III, a protein homologous to mammalian ApoE [56]. ApoLp-III acts endogenously to activate immunity in *Galleria mellonella* by promoting encapsulation, toxic radical formation, and increased phagocytosis by hemocytes [57–59]. ApoLp-III binds to numerous non-self-tissues, including both gram positive [60] and gram negative bacteria [61–63], and has also been shown to induce hemagglutination [64], which suggests it may play a similar and synergistic role in hemocyte aggregation [4]. *Anopheles* ApoLp-III, which is divergent in sequence from Lepidoptera, nonetheless exhibits a similar predicted structure, and is involved in limiting *Plasmodium* infection in the midgut [65].

IC-associated PRRs specific to *B*. *mori* include two C-type lectins, which have been previously characterized in both *B*. *mori* and *M*. *sexta* [66, 67], and the 30 kD Lp proteins, which are well represented in the IC (S7 Fig). Although not extensively functionally characterized, we classify these as PRRs due to their ability to recognize sugars and lipids [68, 69]. A subclass of 30 kD Lps has been reported to bind to ENF peptides such as PSP, but none were found in the IC [31, 32]. The *B*. *mori* IC also contains hemocytin, a large multi-domain protein homologous to human von Willebrand factor and *Drosophila* hemolectin [70, 71]. Studies in *Drosophila* have identified roles for hemolectin in both coagulation and immunity [72–74], and it is also an important component in nodule formation in *B*. *mori* [75].

The LRR-containing LRIM proteins from mosquitos have been studied extensively, particularly in relation to heterodimer formation between *A*. *gambiae* LRIM1 and the APL1 paralogs, and their interaction with the complement-like thioester protein TEP1 [76–78]. This stabilizes the reactive TEP1 and permits opsonization of pathogen surfaces, an important step in *Plasmodium* clearance [79]. No functions are known for the other LRIM proteins found in the IC, but the *A*. *aegypti* LRIM1 is homologous to the *A*. *gambiae* LRIM1, and its incorporation into the melanin-producing IC provides a mechanism for the association of LRIM proteins with melanization in *A*. *gambiae* [80, 81] and *Anopheles quadriannulatus* [82]. The efficacy of pathogen destruction is functionally dependent on the presence of TEP1, and, although we have observed IC-associated TEP1, its presence is inconsistent.

The large array of PRRs found in both ICs suggests it would be profoundly difficult to define their surface binding specificity. In fact, significant effort using various chromatography bead chemistries to mimic non-self failed to clearly determine the surface characteristics that elicit melanin deposition and encapsulation responses [83, 84], for reasons that are now clear. Given these results, a more interesting hypothesis is that the incorporation of certain components within the IC may be dependent on the stimulus that elicits the response. For example, in our experiments, wounding resulted in the incorporation of a subset of the potentially large array of *B*. *mori* 30 kD Lp (S7 Fig) and *A*. *aegypti* LRIM proteins that may be present in the hemolymph [85, 86]. It will be worth investigating whether the introduction of a pathogen produces an altered array of IC components as has been suggested for invertebrates [87], an experimental approach readily accessible by the mass spectrometry-based methods applied here.

*What is the role of the IC-associated SPs*? A major finding in this study is the presence of proteases in the *B*. *mori* and adult *A*. *aegypti* ICs. *B*. *mori* SP3 was first identified by a genome-wide search of clip-domain proteases homologous to other previously identified insect proteases (*Bm*SP124 [88]). There has been no clearly identified function for the SP3 protease or for a highly homologous protease that has been identified in *M*. *sexta* (HP1) from an extensive catalog of clip-domain proteases [89, 90]. HP1 is expressed primarily in hemocytes [89, 91], forms covalent complexes with serpins-4 and -5 upon immune stimulation with bacteria [92], and is also found in a high mass protein complex in *M*. *sexta* [35]. The ability of the native-gel-excised IC to correctly cleave *B*. *mori* Pro-PSP strongly implicates SP3 in the activation of PSP, and the ability of Egf1.0 to inhibit its incorporation into the IC provides additional evidence of its involvement in immunity. Moreover, the association of SP3 with the IC provides a solution to the long-standing mystery as to how PSP can induce hemocytes to encapsulate a non-self-surface. Final confirmation of the function of SP3 will require expression and demonstration of Pro-PSP cleavage activity. However, caution is warranted; like POs, full activation of SP3 may require IC incorporation. Testing whether the *A*. *aegypti* SP (*Aa*CLIPB34) is also involved in hemocyte activation will first require identification of a mosquito cytokine functionally related to PSP. Homologous sequences have been proposed for numerous other species, but have not yet been synthesized and tested in *A*. *aegypti* [24]. Although lacking a known function, *Aa*CLIPB34 is closely related to a set of *Anopheles* SPs (*Ag*CLIPB2,3 and 4) [93]. Knockdowns of *Ag*CLIPB3 and B4 were shown to reduce melanization of parasites and implanted Sephadex beads [94–96]. In contrast, knockdown of *Ag*CLIPB2 did not affect melanization [96], suggesting that it may play a role in hemocyte recruitment in *Anopheles*.

*What induces the formation of the IC and the activation of TGs*? We have demonstrated the formation of a highly integrated IC containing numerous effector proteins related to melanization, coagulation and encapsulation, but what activates these seemingly disparate pathways is unknown. If IC formation were simply an artifact of plasma preparation, then Egf1.0 would not inhibit the incorporation of specific immune components. In addition, we have previously shown in *M*. *sexta* that ICs do not form consistently, but instead are highly dependent on development [5]. In *M*. *sexta*, melanization can be induced by the interaction of pathogen-associated molecular patterns (PAMPs) or β-glucan recognition proteins (βGRP1/2) with hemolymph protease 14 (HP14) [1]. Neither βGRPs nor HP14 were identified in the IC, but *M*. *sexta* Immulectins, homologous to the C-type Lectins found in the IC, have been shown to stimulate PO activity [97]. In *C*. *includens*, *Pseudaletia separata*, and *M*. *sexta*, encapsulation can be induced by ENF peptides, but until now no candidate existed that could explain their activation or localized production. Coagulation occurs in numerous Lepidoptera, but the signal(s) that activate(s) this pathway is also not known. Adding to the complexity is that melanization can be induced by wounding in the absence of added PAMPs, suggesting that there is a self-derived Damage signal that can globally activate the components necessary for IC formation [98]. Much more experimentation is required to answer these challenging questions, but we hypothesize that Damage signals induce assembly of the IC prior to any pathogen interactions, and that the IC then plays an important role in pathogen surveillance.

*How does Egf inhibit melanization*, *IC formation and TG activity*? One of our most intriguing findings is the ability of Egf1.0 to block the incorporation of specific proteins into the *B*. *mori* IC, and to inhibit TG activity. Previous results suggested that Egf1.0 inhibits melanization by inhibiting PAPs [11, 12], which is accomplished by the presence of a P1-P1’ cut site within the catalytic domain (CD) that is identical to PPOs in both sequence (Arg-Phe) and position (residues 51–52). An Egf1.0 mutant containing an Arg^51^ to Ala^51^ substitution confirms this by exhibiting reduced inhibition of PAPs. However, the repeat domain (RD) lacks any sequence homology to PO cascade proteins, yet by itself is quite effective at blocking melanization [12]. This, along with the unexpected finding of Egf1.0 in the IC (Table 2), suggests an entirely different mechanism that does not depend on PAP inhibition. Our results indicate that Egf1.0 may play a broader role in inhibiting immune responses beyond the historical focus on melanization.

An additional and surprising role for Egf1.0 is its ability to inhibit TG activity. To date, no hemolymph TG has been identified in an insect, making a detailed analysis of its activation mechanism and its substrate specificity impossible. However, TG activation in insect hemolymph likely follows one of two pathways exemplified either by the arthropod *Pacifastacus leniusculus* (crayfish), or by mammals. In *P*. *leniusculus*, immune stimulation induces the release of TGs from hemocytes and activation occurs solely through Ca^2+^ binding [2]. In mammals, plasma TG (Factor XIII A chain) is activated by Thrombin-induced proteolysis and Ca^2+^ binding [99]. *B*. *mori* and *M*. *sexta* each possess three genes that are homologous to Factor XIII in the catalytic region, but bear little resemblance to Factor XIII or one another in the N-terminal region where Factor XIII is proteolytically activated. Because active TG cannot be constitutively present in hemolymph, it must either be sequestered or inactive prior to immune stimulation. Therefore, Egf1.0 must either block its release, or prevent its activation, or directly inhibit it post-activation. Caution is warranted in this interpretation because inhibition could be due to a lack of TG substrates such as POs and storage proteins in the IC. However, our pull-down experiments of biotin-tagged proteins showed numerous TG targets within the IC, including proteins whose incorporation is not inhibited by Egf1.0 (Alps, hemocytin and 30 kD Lp proteins) (S1–S3 Tables). These findings suggest that sufficient TG substrates remain in the Egf1.0-treated IC to demonstrate TG activity. This hypothesis highlights a much stronger case for Egf1.0-induced inhibition of TG activity, but still begs the question as to the mechanism. While activation through the PO cascade may provide the most parsimonious explanation, it is troublesome due to the apparent lack of proteolytic activation sites. Clearly, more experiments are required to discover the signal(s) that induces formation of the IC, to dissect its structure, to understand the role of TG in stabilizing it, and to determine the mechanism by which Egf1.0 inhibits both processes. The results presented here begin to address these major questions in insect immunity, and open promising new avenues of research.

## Materials and methods

### Insects and plasma collection

*B*. *mori* and *M*. *sexta* larvae were reared on artificial diet at 27°C and a 16 h light: 8 h dark photoperiod as previously described [10, 100]. Hemolymph was collected by narcotizing 4^th^ or 5^th^ instars with CO_2_ and then bleeding each larva from a cut proleg into a 1.5 mL Eppendorf tube on ice [4]. Glutathione (GSH, 5 mM final concentration) and phenylthiourea (PTU, 0.1 mM final concentration) were added immediately after hemolymph collection to prevent melanization [101]. However, they do not block formation of the IC, which requires additional treatment with the inhibitor Egf1.0 (30 μL of a 0.1 mg/mL Egf1.0 solution to 100 μL of hemolymph) [6]. Treated hemolymph samples were immediately centrifuged at 16,000 x *g* for 30 sec to pellet hemocytes and the resulting plasma supernatant was transferred to a new centrifuge tube for experimental use. The UGAL strain of *A*. *aegypti* was reared in an insectary at 27°C and 60% relative humidity using a 16 h light: 8 h dark photoperiod. Larvae were fed a standard diet consisting of finely ground rat chow (Purina): lactalbumin: brewers yeast (1:1:1) in open aluminum rearing pans containing distilled water. Pupae were transferred from larval rearing pans to plastic cages for emergence, which produced large cohorts of similar sized adults that were fed water supplemented with 5% sucrose (wt/vol) on day 2. To obtain hemolymph, pupae and non-blood-fed adults were cold anesthetized and then perfused with 2.5 μl of TRIS-buffered saline (TBS) containing 100 μM PTU by injection into the thorax using a pulled capillary pipet and collection through a hole in the last abdominal segment. Pooled samples were centrifuged at 9k rpm for 10 min at 4°C to remove hemocytes and other tissues.

### Preparation of substrate-free plasma and melanization assay

Plasma samples were diluted 10-fold with TBS and concentrated to their original volume on a 30 kDa centrifugal filter (Amicon Ultra, Ultracel-30k). This procedure was repeated three times, providing an approximately 1000-fold dilution of constituents less than 30 kDa. Centrifugation separated the plasma into a viscous lower layer, and a clear upper layer, which were removed separately and assayed for activity. Melanin production was measured as described previously [6]. Briefly, 40 μL of substrate-free plasma was combined with 40 μL TBS and 20 μL of a 2.5 mM tyrosine (Tyr) solution. Melanin formation was monitored by absorbance at 470 nm at 3 min intervals using a BioTek Synergy 4 plate reader. The melanization inhibitor Egf1.0 was prepared as described previously [12].

### Native gels, SDS-PAGE, immunoblotting, affinity-blotting, and mass spectrometry

Plasma samples for native gel analysis were prepared by combining 10 μL of plasma containing GSH and PTU ±Egf1.0 with 5 μL TRIS-Glycine running buffer pH 8 and 5 μL 4x native-PAGE sample buffer (ThermoFisher). Twenty μL was loaded onto duplicate lanes of a pre-cast 4–20% gel (Lonza) and run in TRIS-glycine running buffer for 3 h at 80V in a 4°C cold room. Melanizing activity was determined by incubating gel lanes in a 0.5 mM solution of Tyr or dopamine (DA) for 2 h, after which they were rinsed in water and photographed using visible light transmission.

For protein identification by MS, duplicate lanes were Coomassie-stained, and the protein bands at the gel/well interface corresponding to the melanization activity were excised and treated with 10 mM dithiothreitol (DTT) for 1 h at 37°C. The DTT solution was removed and the bands treated for 30 min with 100 mM iodoacetamide in a light free environment before digestion with Trypsin overnight at 37°C. Peptides were extracted with acetonitrile/H_2_0 (50:50 with 1% trifluoroacetic acid) and separated using a Thermo Acclaim Pepmap 100 C8 column (75μm x 15 cm) on an Easy-nLC-II (ThermoScientific). Peptides were eluted at 500 nL/min using a multi-step gradient of solvent A (0.1% formic acid (FA) in H_2_O) and solvent B (0.1% FA in acetonitrile, 10%-40% for 70 min, 40%-95% for 20 min, 95%-95% for 5 min). The eluent was analyzed using an Orbitrap Elite mass spectrometer (ThermoScientific). The MS data were processed by Proteome Discoverer 1.4 (ThermoScientific) in conjunction with Matrix Science MASCOT 2.3 software.

For SDS-PAGE, 2 μL of plasma were combined with 10 μL 4x NuPAGE sample buffer (ThermoFisher), 4 μL 500 mM DTT (Sigma) and 24 μL water and boiled for 5 min. Sixteen μL samples were loaded onto 4–20% SDS-PAGE gels (Lonza) along with molecular weight standards (Fermentas, PageRuler Plus). For immunoblotting, proteins were transferred onto PVDF (EMD Millipore, Immobilon-P), blocked in 5% non-fat powdered milk in PBST (PBS with 2% Tween-20), and probed overnight at 4°C with *B*.*mori*-specific antibodies (20,000-fold dilution) that recognize both the precursor and processed forms of PPO, PPO1, PPO2, and Storage Protein 1 (SP1) [6]. After washing 3x with PBST, the blot was incubated with a goat anti-rabbit secondary antibody coupled to HRP (ThermoFisher, 25,000-fold dilution) for 4 h at 4°C. Blots were then washed 3x with PBST followed by visualization using a chemiluminescent substrate (GE Healthcare, ECL Advance) as described [11]. For affinity-blotting, biotin-containing samples were treated in a similar manner as the immunoblots, but were developed with Streptavidin-Horseradish Peroxidase (SAv-HRP, ThermoFisher). The digital images of both the native gels and the affinity/immunoblots were processed using Adobe Photoshop. Levels were adjusted uniformly and the whole image was sharpened for clarity. No other image processing was performed.

### *B*. *mori* Pro-PSP expression and protease processing

Total RNA from *B*. *mori* was extracted from a 5^th^ instar larva using TRIzol (ThermoFisher). First strand cDNA was synthesized using a Transcriptor High Fidelity cDNA synthesis Kit (Roche). The full-length ORF was amplified using the primers 5’-ATG AAG TGT TCG GTT TTT ATT TTG TGT TG-3’ (forward) and 5’-TCA AAA GGT AGG TTT ACA TCT GCC AT-3’(reverse). The amplified PCR product was cloned into a TA cloning vector (ThermoFisher) and used as a template to generate a fragment containing Ek/LIC overhangs employing the primers 5'-GAC GAC GAC AAG ATG GGA GTA AAC GGT TTC TTC AAT G-3' (forward) and 5’-GAG GAG AAG CCC GGT TCA AAA GGT AGG TTT ACA TCT GC-3' (reverse). After treatment with T4 DNA polymerase, the amplified fragment was cloned into a pET-30 vector (Novagen). The constructed vectors were confirmed by DNA sequencing. For bacterial expression, *E*. *coli* strain BL21 (DE3) was transformed with the construct pET30-rBmPro-PSP. The transformed cells were cultured in SOC medium (0.5% Yeast Extract, 2% Tryptone, 10 mM NaCl, 2.5 mM KCl, 10 mM MgCl_2_, 10 mM MgSO_4_, 20 mM Glucose) supplemented with 10 μg/mL of kanamycin. Bacteria were grown to an O.D. of 1.0 at 37°C and then induced by isopropyl-β-D-thiogalactopyranoside (IPTG) at a 0.1 mM final concentration. The culture was incubated for another 16 h at 16°C, after which the cells were harvested by centrifugation at 5000 x *g* for 10 min. The pellet was stored at -20°C until further use. To obtain Pro-PSP, the bacterial pellet from a 0.8 L culture was resuspended in 40 mL of lysis buffer (50 mM NaH_2_PO_4_, 300 mM NaCl, 10 mM imidazole, pH 8.0). The resuspended cells were incubated on a rotator at 22°C for 1 h after adding lysozyme to 1mg/ml and then sonicated with twenty 1 s bursts at 200 W (Branson, USA). After centrifugation of the lysate at 10,000 x *g* for 20 min, the supernatant was bound to Ni-NTA matrix (5 Prime) pre-equilibrated with lysis buffer. Proteins were eluted with three column volumes of elution buffer (50 mM NaH_2_PO_4_, 300 mM NaCl, 300 mM imidazole) after washing with washing buffer (50 mM NaH_2_PO_4_, 300 mM NaCl, 20 mM imidazole). The eluted proteins also were desalted and concentrated by Centricon 10 (Millipore, USA). To assess Pro-PSP processing by the IC, the narrow bands at the gel/well interface of native gels were excised and placed into Eppendorf tubes containing 25 μL of a 0.1 mg/mL solution of Pro-PSP in TBS. Tubes were incubated at 22°C for 2 h, after which the supernatants were removed and tested for proteolytic processing of Pro-PSP by SDS-PAGE and mass spectrometry (MS). SDS-PAGE samples were prepared and run as described above, except that proteins were stained with Oriole (Bio-Rad). The mass of the peptide cleaved from Pro-PSP was determined by LC-MS using an Applied Biosystems 140B solvent delivery system with a Waters Micromass Q-TOF Micro MS. The sample was separated on a Thermo Hypersil-Keystone Biobasic-4 (1 mm x 150 mm) column using a gradient of solvent A (1% FA in H_2_O) and solvent B (1% FA in acetonitrile, 3%-100% for 35 min) at a flow rate of 50 μL/min.

## Supporting information

S1 Fig Synthesis of novel TG substrates. KGC-B, GGKGC-B and RhB-GGKGC-B were synthesized by standard methods as described previously (Clark et al., JBC 2004:279, p. 33246) except that RhB-GGKGC-B was manually acylated at the N-terminus with rhodamine B (Sigma) on-resin. After cleavage and purification, the Cys sulfhydryl on each peptide was derivatized with thiol-reactive EZ-Link HPDP-Biotin (ThermoFisher). The resultant disulfide-containing peptide was again purified, and the mass confirmed by MALDI-TOF MS. The disulfide-linked biotin provides an orthogonal release mechanism from avidin-based affinity reagents, which can be effected either by boiling in SDS to denature the avidin (which leaves biotin attached to the protein), or by reduction of the disulfide (which removes biotin from the protein). The rhodamine-capped version provides an alternative means of visualizing the cross-linked proteins if desired, but another affinity tag or fluorescent indicator could also be used.
(TIF)

S2 Fig Labeling efficiency of B-cad and KGC-B. Plasma was prepared as described in the Methods, and B-cad and KGC-B were added to a final concentration of 4 mM. Samples were incubated at 22°C for 2 h, boiled in 4x NuPAGE sample buffer (ThermoFisher) without DTT, and then separated by SDS-PAGE using a 4–20% gradient gel (Lonza). The gel was transferred and affinity-blotted as in Fig 3. The exposure of the B-cad and KGC-B lanes was adjusted equally and uniformly by Adobe Photoshop using Levels. The B-cad* lane is an electronic copy of the B-cad lane that has been darkened using Levels in Adobe Photoshop in order to visualize the faintly labeled proteins. No other adjustments were made.
(TIF)

S3 Fig Labeling efficiency of (A) KGC-B, (B) GGKGC-B, and (C) RhB-GGKGC-B. *Chrysodeixis (Pseudoplusia) includens* plasma was prepared as described in the Materials and Methods, labeled with each of the TG substrate peptides, run on SDS-PAGE, and affinity-blotted as in S2 Fig. Numbers refer to the volume loaded onto the gel.
(TIF)

S4 Fig Affinity-purification of KGC-B-labeled TG substrates and identification by MS. Plasma was prepared and incubated with 2 mM KGC-B for 40 min at 22°C. The plasma was then dialyzed twice for twelve hours using 1 L TBS at 4°C with SnakeSkin Dialysis Tubing (10 kD MWCO, ThermoFisher) to remove excess substrate. Biotin-containing proteins were affinity-purified by StreptAvidin UltraLInk Resin (ThermoFisher). Resin slurry (0.9 ml) was washed 3x with TBS, added to 3 ml of the dialyzed plasma, and then mixed at 22°C for 2.5 h. The resin was washed by adding 14 mL of TBS and vigorously vortexing the mixture, after which it was centrifuged and the supernatant removed. This procedure was repeated three times. The washed resin was resuspended in 550 μL TBS and 60 μL 4x NuPAGE sample buffer (ThermoFisher) and then boiled for 10 min with frequent vortexing to keep the resin suspended. The sample was cooled on ice, the resin pelleted, and the supernatant removed. The eluted proteins were run on SDS-PAGE ± DTT as described (S2 Fig), and stained with Oriole (Bio-Rad). The labeled proteins were excised and identified by MS.
(TIF)

S5 Fig Egf1.0 inhibits the formation and surface binding of the IC. Silica chromatography beads were washed in TBS and then added to freshly prepared plasma ±Egf1.0. The beads were mixed for 1 h and then washed 4x with TBS. A 0.5 mM solution of Tyr in TBS was then added to the beads to allow melanin formation. After 30 min, the beads were washed again to remove substrate and visible light pictures were taken using a dissecting microscope.
(TIF)

S6 Fig Alignment of the catalytic region of the *Aedes aegypti* IC-associated SP with SPHs. The catalytic triad is shown encased by boxes, while the arrow indicates the lack of an active Ser in all but AAEL000028. Alignments were generated by CLC Sequence Viewer 7.7.
(TIF)

S7 Fig Cladogram of *B*. *mori* 30 kD Lp proteins. Sequences determined by Zhang et al. [31] were retrieved from the SilkDB genome database and plotted as a cladogram by CLC Sequence Viewer Version 7.6.1 (QIAGEN Aarhus A/S). Components identified in the IC and by affinity purification are labeled. Members predicted to bind ENF-peptides are highlighted. The cladogram was generated by CLC Sequence Viewer 7.7.
(TIF)

S1 Table Identification of biotin-tagged apolipophorins, hemocytin, and hexamerins by LC-MS/ MS. Data presented are the percent coverage of the protein. M_r_ values are determined from mobility measurements relative to the protein standards. M_r_ (calculated) values are nominal molecular weights obtained from sequence data. (a) Apolipophorins (combined mass prior to post-translational processing). (b) Hypothetical proteins homologous to apolipophorins (combined mass prior to post-translational processing). (c) Storage proteins 1 and 2. (d) A hexamerin homologous to the riboflavin-binding hexamerin from Hyalophora (Magee et al., 1994).
(DOCX)

S2 Table Identification of biotin-tagged putative enzymes and protease inhibitors by LC-MS/MS. (a) Serine-protease-like protein 2. (b) Carboxylesterase. (c) Glu-carboxy-peptidase. (d) Trypsin inhibitor with limited homology to a von Willebrand factor type A domain. (e) Serpins nomenclature (100)
(DOCX)

S3 Table Identification of biotin-tagged 30 kD Lp proteins by LC-MS/MS. *See reference [31].
(DOCX)
