# Supplementary figures and images for: Bombyx mori and Aedes aegypti form multi-functional immune complexes that integrate pattern recognition, melanization, coagulants, and hemocyte recruitment
Source: PLoS One. 2017 Feb 15;12(2):e0171447. doi: 10.1371/journal.pone.0171447 (PMC5310873; doi:10.1371/journal.pone.0171447)

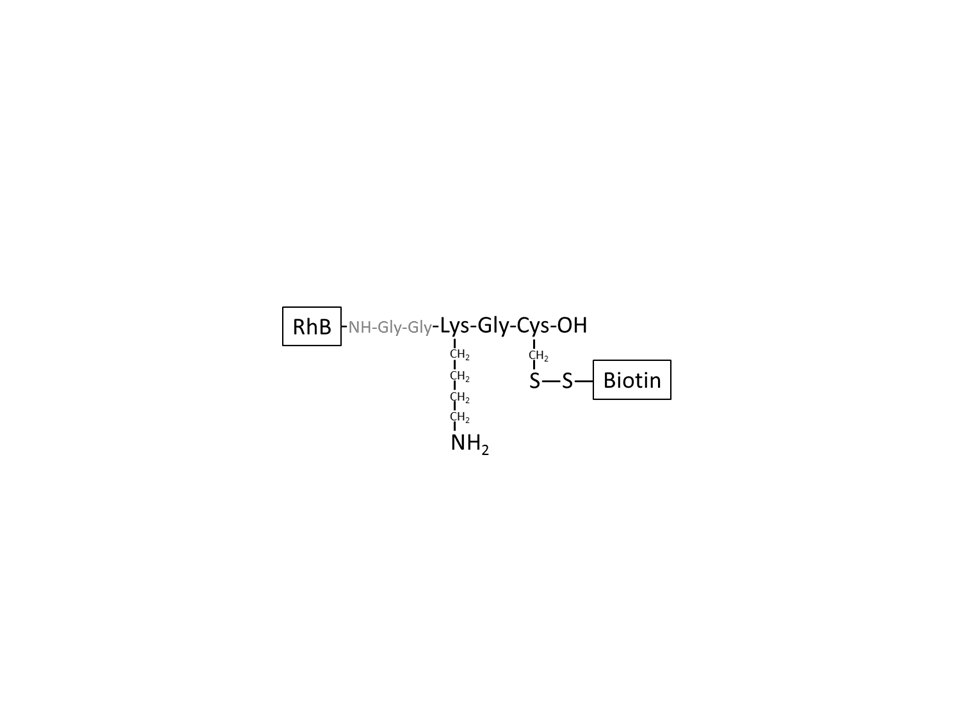

Supplement: S1 Fig — KGC-B, GGKGC-B and RhB-GGKGC-B were synthesized by standard methods as described previously (Clark et al., JBC 2004:279, p. 33246) except that RhB-GGKGC-B was manually acylated at the N-terminus with rhodamine B (Sigma) on-resin. After cleavage and purification, the Cys sulfhydryl on each peptide was derivatized with thiol-reactive EZ-Link HPDP-Biotin (ThermoFisher). The resultant disulfide-containing peptide was again purified, and the mass confirmed by MALDI-TOF MS. The disulfide-linked biotin provides an orthogonal release mechanism from avidin-based affinity reagents, which can be effected either by boiling in SDS to denature the avidin (which leaves biotin attached to the protein), or by reduction of the disulfide (which removes biotin from the protein). The rhodamine-capped version provides an alternative means of visualizing the cross-linked proteins if desired, but another affinity tag or fluorescent indicator could also be used. (TIF) [file pone.0171447.s001.TIF]

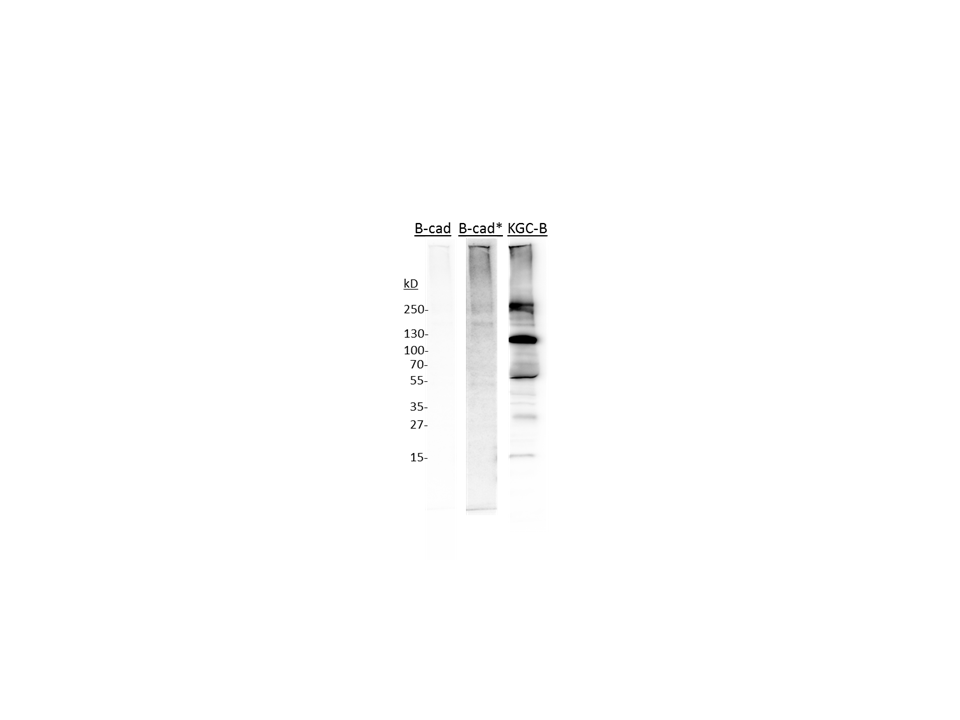

Supplement: S2 Fig — Plasma was prepared as described in the Methods, and B-cad and KGC-B were added to a final concentration of 4 mM. Samples were incubated at 22°C for 2 h, boiled in 4x NuPAGE sample buffer (ThermoFisher) without DTT, and then separated by SDS-PAGE using a 4–20% gradient gel (Lonza). The gel was transferred and affinity-blotted as in Fig 3. The exposure of the B-cad and KGC-B lanes was adjusted equally and uniformly by Adobe Photoshop using Levels. The B-cad* lane is an electronic copy of the B-cad lane that has been darkened using Levels in Adobe Photoshop in order to visualize the faintly labeled proteins. No other adjustments were made. (TIF) [file pone.0171447.s002.TIF]

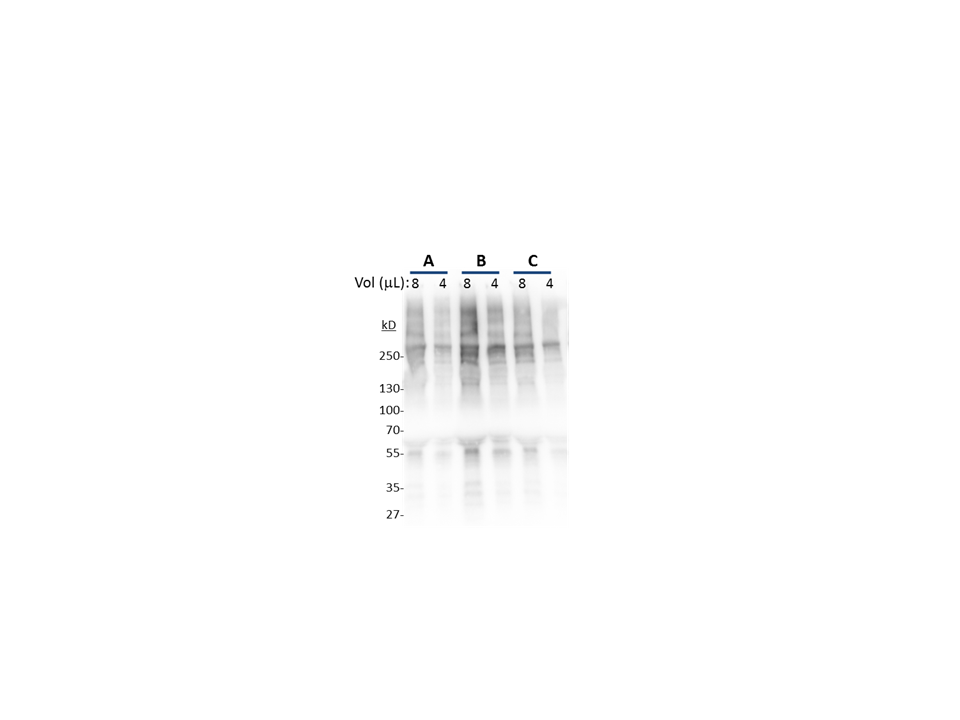

Supplement: S3 Fig — Chrysodeixis (Pseudoplusia) includens plasma was prepared as described in the Materials and Methods, labeled with each of the TG substrate peptides, run on SDS-PAGE, and affinity-blotted as in S2 Fig. Numbers refer to the volume loaded onto the gel. (TIF) [file pone.0171447.s003.TIF]

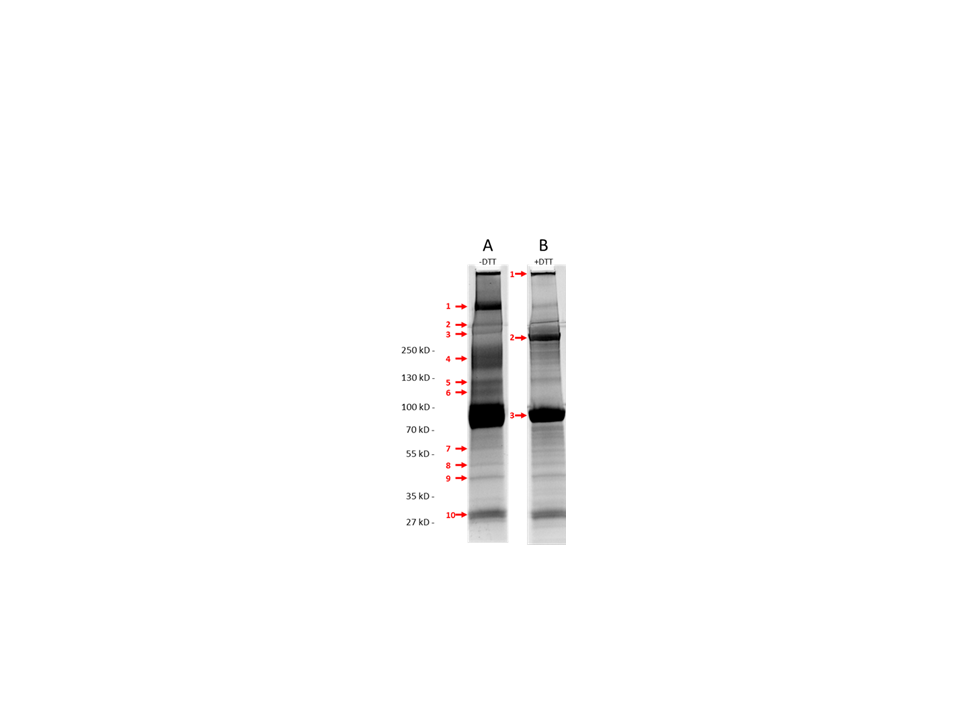

Supplement: S4 Fig — Plasma was prepared and incubated with 2 mM KGC-B for 40 min at 22°C. The plasma was then dialyzed twice for twelve hours using 1 L TBS at 4°C with SnakeSkin Dialysis Tubing (10 kD MWCO, ThermoFisher) to remove excess substrate. Biotin-containing proteins were affinity-purified by StreptAvidin UltraLInk Resin (ThermoFisher). Resin slurry (0.9 ml) was washed 3x with TBS, added to 3 ml of the dialyzed plasma, and then mixed at 22°C for 2.5 h. The resin was washed by adding 14 mL of TBS and vigorously vortexing the mixture, after which it was centrifuged and the supernatant removed. This procedure was repeated three times. The washed resin was resuspended in 550 μL TBS and 60 μL 4x NuPAGE sample buffer (ThermoFisher) and then boiled for 10 min with frequent vortexing to keep the resin suspended. The sample was cooled on ice, the resin pelleted, and the supernatant removed. The eluted proteins were run on SDS-PAGE ± DTT as described (S2 Fig), and stained with Oriole (Bio-Rad). The labeled proteins were excised and identified by MS. (TIF) [file pone.0171447.s004.TIF]

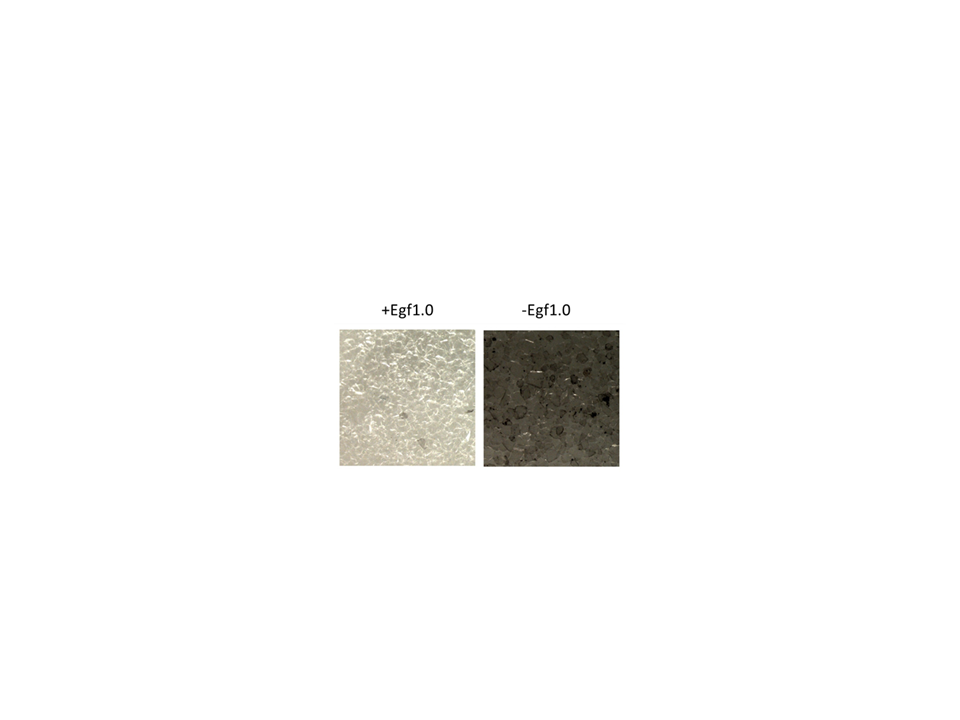

Supplement: S5 Fig — Silica chromatography beads were washed in TBS and then added to freshly prepared plasma ±Egf1.0. The beads were mixed for 1 h and then washed 4x with TBS. A 0.5 mM solution of Tyr in TBS was then added to the beads to allow melanin formation. After 30 min, the beads were washed again to remove substrate and visible light pictures were taken using a dissecting microscope. (TIF) [file pone.0171447.s005.TIF]

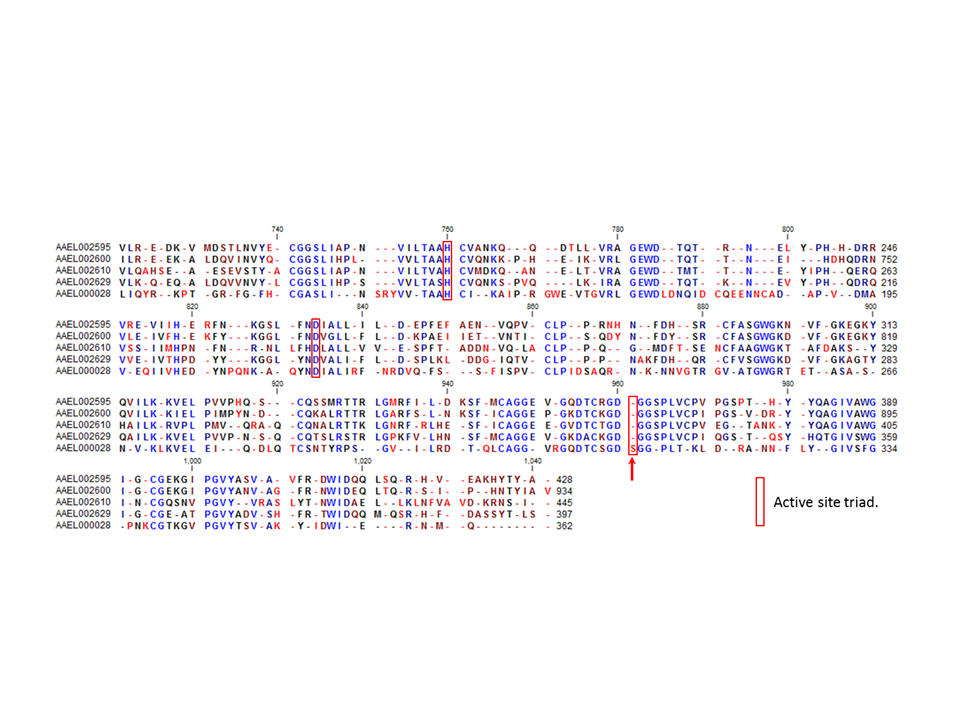

Supplement: S6 Fig — The catalytic triad is shown encased by boxes, while the arrow indicates the lack of an active Ser in all but AAEL000028. Alignments were generated by CLC Sequence Viewer 7.7. (TIF) [file pone.0171447.s006.TIF]

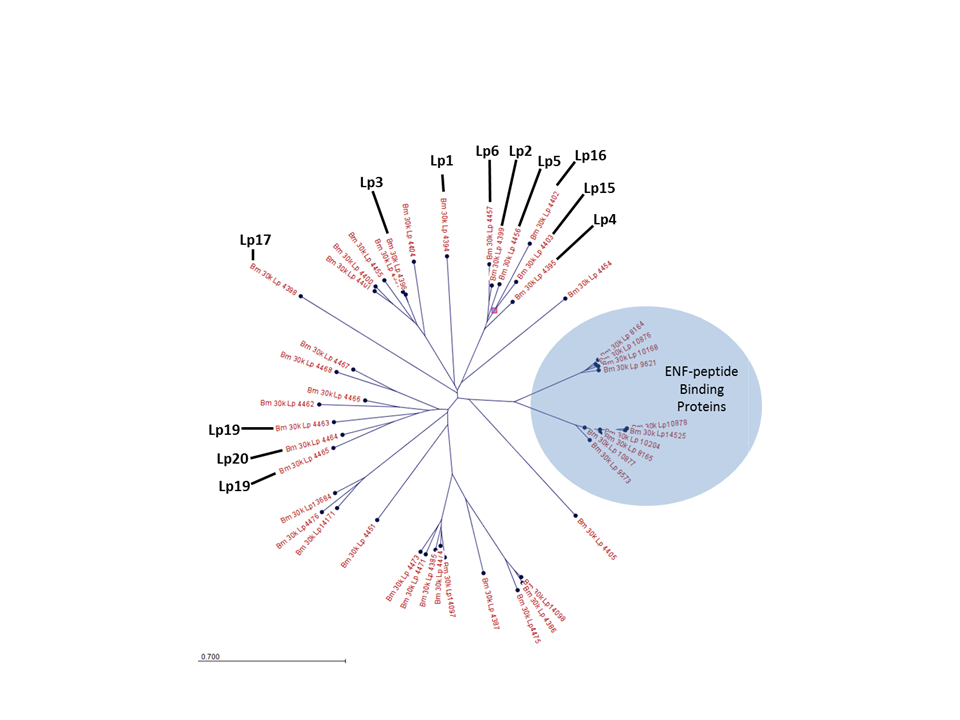

Supplement: S7 Fig — Sequences determined by Zhang et al. [31] were retrieved from the SilkDB genome database and plotted as a cladogram by CLC Sequence Viewer Version 7.6.1 (QIAGEN Aarhus A/S). Components identified in the IC and by affinity purification are labeled. Members predicted to bind ENF-peptides are highlighted. The cladogram was generated by CLC Sequence Viewer 7.7. (TIF) [file pone.0171447.s007.TIF]
