# Supplementary material for: Bombyx mori and Aedes aegypti form multi-functional immune complexes that integrate pattern recognition, melanization, coagulants, and hemocyte recruitment
Source: PLoS One. 2017 Feb 15;12(2):e0171447. doi: 10.1371/journal.pone.0171447 (PMC5310873; doi:10.1371/journal.pone.0171447)
Supplement: S1 Table — Data presented are the percent coverage of the protein. Mr values are determined from mobility measurements relative to the protein standards. Mr (calculated) values are nominal molecular weights obtained from sequence data. (a) Apolipophorins (combined mass prior to post-translational processing). (b) Hypothetical proteins homologous to apolipophorins (combined mass prior to post-translational processing). (c) Storage proteins 1 and 2. (d) A hexamerin homologous to the riboflavin-binding hexamerin from Hyalophora (Magee et al., 1994). (DOCX) [file pone.0171447.s008.docx]

| **Protein ID**🡪 | | **pAlpII**  **(a)** | **pAlpI**  **(a)** | **H.P. 1**  **(b)** | **H.P. 2**  **(b)** | **Hemocytin** | **SP1**  **(c)** | **SP2**  **(c)** | **Aryl-**  **phorin** | **Hexamerin**  **(d)** | **PO1** | **PO2** |
| --- | --- | --- | --- | --- | --- | --- | --- | --- | --- | --- | --- | --- |
| **SilkDB (BGIBMGA0…)** | | 13341 | 13342 | 13893 | 13894 | 08859 | 11266 | 09028 | 09027 | 08859 | 12763 | 13115 |
| **M_r_ (calculated)**🡪 | | 290kD | | 293kD | | 280kD | 87kD | 83kD | 83kD | 87kD | 79kD | 80kD |
| **Band** | M_r_ |  |  |  |  |  |  |  |  |  |  |  |
| **A1** | **351** | **6.8** | **63** | **2.7** | **4.2** | **9.8** | **8.3** |  |  | **10** |  |  |
| **A2** | **280** | **17** | **72** | **8.1** | **17** | **11** | **25** |  |  | **11** |  |  |
| **A3** | **251** |  | **70** |  |  | **6.1** | **11** |  |  | **6.0** |  |  |
| **A4** | **188** | **29** | **58** |  |  | **18** | **34** | **11** | **13** | **18** |  | **6.0** |
| **A5** | **138** | **13** | **45** |  |  | **8.8** | **9** |  |  | **8.8** |  |  |
| **A6** | **122** | **4.0** | **31** |  |  | **7.9** | **9.2** | **2.7** | **7.6** | **7.9** |  |  |
| **A7** | **61** | **11** | **11** |  |  | **12.5** | **9.1** | **9.5** | **14** | **13** | **4.5** | **9.0** |
| **A8** | **50** | **8.5** | **6.0** |  |  | **6.9** | **8.7** |  | **6.5** | **6.9** |  |  |
| **A9** | **42** | **7.4** | **6.2** |  |  |  | **8.3** | **2.7** | **5.0** |  |  |  |
| **A10** | **27** |  |  |  |  |  | **2.8** |  | **4.5** |  |  |  |
|  |  |  |  |  |  |  |  |  |  |  |  |  |
| **B1** | **Well/gel** |  | **39** |  |  |  | **13** |  |  |  |  |  |
| **B2** | **243** | **9.0** | **67** |  |  |  | **56** |  |  |  |  | **5.1** |
| **B3** | **92** | **38** | **9.5** | **3.9** |  | **35** | **55** | **25** | **30** | **35** |  |  |
|  |  |  |  |  |  |  |  |  |  |  |  |  |
