# Supplementary material for: Bombyx mori and Aedes aegypti form multi-functional immune complexes that integrate pattern recognition, melanization, coagulants, and hemocyte recruitment
Source: PLoS One. 2017 Feb 15;12(2):e0171447. doi: 10.1371/journal.pone.0171447 (PMC5310873; doi:10.1371/journal.pone.0171447)
Supplement: S2 Table — (a) Serine-protease-like protein 2. (b) Carboxylesterase. (c) Glu-carboxy-peptidase. (d) Trypsin inhibitor with limited homology to a von Willebrand factor type A domain. (e) Serpins nomenclature (100) (DOCX) [file pone.0171447.s009.docx]

| **Protein ID**🡪 | | **SP-LP**  **(a)** | **CE**  **(b)** | **GCP**  **(c)** | **T.I.**  **(d)** | **Serpin 9**  **(CI-8A) (e)** | **Serpin 1 (e)** |
| --- | --- | --- | --- | --- | --- | --- | --- |
| **SilkDB (BGIBMGA0…)** | | 03688 | 06456 | 07728 | 07558 | 01983 | 09953 |
| **M_r_ (calculated)**🡪 | | 40kD | 75kD | 52kD | 84kD | 42/38kD | 38kD |
| **Band** | M_r_ |  |  |  |  |  |  |
| **A1** | **351** |  |  |  | **4.9** |  |  |
| **A2** | **280** |  |  |  | **9.5** |  |  |
| **A3** | **251** |  |  |  | **9.5** |  |  |
| **A4** | **188** |  |  |  | **10** |  |  |
| **A5** | **138** | **16** |  |  | **40** |  |  |
| **A6** | **122** |  | **4.4** |  | **19** |  |  |
| **A7** | **61** |  |  | **18** |  |  |  |
| **A8** | **50** | **7.2** |  |  | **5.6** | **46** |  |
| **A9** | **42** | **37** |  |  |  | **19** | **43** |
| **A10** | **27** | **7.2** | **16** |  |  |  |  |
|  |  |  |  |  |  |  |  |
| **B1** | **Well/**  **gel** |  |  |  |  |  |  |
| **B2** | **243** |  |  |  |  |  |  |
| **B3** | **92** |  |  |  |  |  |  |
