# Supplementary material for: Bombyx mori and Aedes aegypti form multi-functional immune complexes that integrate pattern recognition, melanization, coagulants, and hemocyte recruitment
Source: PLoS One. 2017 Feb 15;12(2):e0171447. doi: 10.1371/journal.pone.0171447 (PMC5310873; doi:10.1371/journal.pone.0171447)
Supplement: S3 Table — *See reference [31]. (DOCX) [file pone.0171447.s010.docx]

| **SilkDB (BGIBMGA0…)** | | **4394** | **4395** | **4396** | **4398** | **4399** | **4402** | **4456** | **4457** | **4463** | **4464** | **4465** |
| --- | --- | --- | --- | --- | --- | --- | --- | --- | --- | --- | --- | --- |
| **Nomenclature***🡪 | | **BmLp**  **1** | **BmLp**  **4** | **BmLp**  **3** | **BmLp**  **17** | **BmLp2** | **BmLp**  **16** | **BmLp5** | **BmLp6** | **BmLp**  **19** | **BmLp**  **20** | **BmLp**  **21** |
| **Band** | M_r_ |  |  |  |  |  |  |  |  |  |  |  |
| **A1** | **351** |  | **19** |  |  |  |  |  |  |  |  |  |
| **A2** | **280** | **7.0** | **32** |  |  |  |  |  |  |  |  |  |
| **A3** | **251** |  | **19** |  |  |  |  |  |  |  |  |  |
| **A4** | **188** | **10** | **22** |  |  |  |  |  |  |  |  |  |
| **A5** | **138** | **20** | **50** |  |  | **28** |  |  |  |  |  |  |
| **A6** | **122** | **20** | **53** |  |  | **25** |  |  |  |  |  |  |
| **A7** | **61** | **18** | **53** |  |  | **28** |  |  |  |  |  |  |
| **A8** | **50** | **8.2** | **35** |  |  |  |  |  |  |  |  |  |
| **A9** | **42** | **17** | **41** |  |  | **25** |  |  |  |  |  |  |
| **A10** | **27** | **39** | **64** | **5.9** | **17** | **59** | **8.9** | **35** | **42** | **2.7** | **39** | **33** |
|  |  |  |  |  |  |  |  |  |  |  |  |  |
| **B1** | **Well/gel** |  |  |  |  |  |  |  |  |  |  |  |
| **B2** | **243** |  |  |  |  |  |  |  |  |  |  |  |
| **B3** | **92** |  |  |  |  |  |  |  |  |  |  |  |
|  |  |  |  |  |  |  |  |  |  |  |  |  |
